# Complex nature of SNP genotype effects on gene expression in primary human leucocytes

**DOI:** 10.1186/1755-8794-2-1

**Published:** 2009-01-07

**Authors:** Graham A Heap, Gosia Trynka, Ritsert C Jansen, Marcel Bruinenberg, Morris A Swertz, Lotte C Dinesen, Karen A Hunt, Cisca Wijmenga, David A vanHeel, Lude Franke

**Affiliations:** 1 Institute of Cell and Molecular Science, Barts and The London School of Medicine and Dentistry, London, E1 2AT, UK; 2 Genetics Department, University Medical Centre Groningen, University of Groningen, 9700 RB Groningen, the Netherlands; 3 Complex Genetics Section, DBG-Department of Medical Genetics, University Medical Centre Utrecht, 3584 CG Utrecht, the Netherlands; 4 Groningen Bioinformatics Centre, Groningen Biomolecular Sciences and Biotechnology Institute, University of Groningen, Kerklaan 30, NL-9751 NN Haren, the Netherlands; 5 Gastroenterology Unit, University of Oxford, Oxford OX3 7BN, UK

## Abstract

**Background:**

Genome wide association studies have been hugely successful in identifying disease risk variants, yet most variants do not lead to coding changes and how variants influence biological function is usually unknown.

**Methods:**

We correlated gene expression and genetic variation in untouched primary leucocytes (n = 110) from individuals with celiac disease – a common condition with multiple risk variants identified. We compared our observations with an EBV-transformed HapMap B cell line dataset (n = 90), and performed a meta-analysis to increase power to detect non-tissue specific effects.

**Results:**

In celiac peripheral blood, 2,315 SNP variants influenced gene expression at 765 different transcripts (< 250 kb from SNP, at FDR = 0.05, *cis *expression quantitative trait loci, eQTLs). 135 of the detected SNP-probe effects (reflecting 51 unique probes) were also detected in a HapMap B cell line published dataset, all with effects in the same allelic direction. Overall gene expression differences within the two datasets predominantly explain the limited overlap in observed *cis*-eQTLs. Celiac associated risk variants from two regions, containing genes *IL18RAP *and *CCR3*, showed significant *cis *genotype-expression correlations in the peripheral blood but not in the B cell line datasets. We identified 14 genes where a SNP affected the expression of different probes within the same gene, but in opposite allelic directions. By incorporating genetic variation in co-expression analyses, functional relationships between genes can be more significantly detected.

**Conclusion:**

In conclusion, the complex nature of genotypic effects in human populations makes the use of a relevant tissue, large datasets, and analysis of different exons essential to enable the identification of the function for many genetic risk variants in common diseases.

## Background

Human gene expression levels have a strong heritable component [1-4]. At some genes, the variance in gene expression levels is an order of magnitude greater between unrelated individuals, than between identical twins [5]. Quantitative mRNA levels are key regulators of phenotype and represent a link between genetic variation and phenotypic alterations. A term first introduced by Jansen & Nap [6], *genetical genomics *aims to identify the genetic variants that affect gene expression. By treating gene expression as a quantitative trait it is possible to correlate gene transcript expression levels with genomic locations such that expression quantitative trait loci (eQTLs) can be identified [7]. In the human genome, *cis *associations, where a genetic variant affects a transcript that maps to the same locus, have been predominantly reported [3,8]. *Trans *effects, where the genetic variant is distant to the transcript loci, are much harder to convincingly identify due to inherent multiple testing problems. Analysis of *trans *effects involves several magnitudes more statistical tests than for *cis *effects. Although individual studies have reported human *trans *associations, no effects have been convincingly replicated in multiple studies identified for the same transcript and variant [1,9,10].

Quantitative transcript expression and genotype relationships can be investigated via linkage or association based methodologies. Linkage studies use a genome wide series of markers in recombinant in-bred lines or families, to follow the heritability of a trait, whilst association studies usually compare large number of single nucleotide polymorphisms (SNPs) to transcript levels from unrelated individuals. Despite its extensive use in plant [11,12], mouse [13,14] and rat [15] models, transcript expression and genotype correlation studies have only recently been performed in humans [3,5,8].

Human association studies have centered on RNA obtained from leukocytes, predominantly Epstein-Barr virus (EBV) transformed B cell lines from HapMap individuals [3] or unrelated trios [1]. A large scale genetical genomics linkage study has recently identified more than 1000 *cis *regulatory loci across the genome, in primary cryopreserved human leukocytes [2], although these are broad genomic regions rather than variants in more precisely defined LD blocks. However, the former studies are limited by their ability to assess transcripts that are significantly expressed at the point of RNA isolation. Cell extraction methods, cryopreservation and EBV transformation all affect individual mRNA expression levels. These variations in RNA analysis make the choice of analysis tissue of paramount importance.

Here we present an association based genetical genomics study using primary cell RNA from peripheral blood sampled from patients in remission from an immune-mediated disease. Immediate RNA preservation during blood sampling (using the PAXgene system) represents nearly *in vivo *human physiological gene expression. Celiac disease is a common (1% prevalence), inflammatory condition of the small intestine induced by intake of gluten in wheat, rye and barley. A strong genetic component has been established for disease with a monozygotic concordance of 75% [16] and 90% of cases possessing the HLA haplotype HLA-DQ2.5 [17] and the remainder mostly have HLA-DQ8 [18]. Despite the role of the HLA, the risk of disease is still greater in HLA matched monozygotic twins compared to HLA matched dizygotic twins [19]. We recently performed a genome wide association and replication study using single nucleotide polymorphisms (SNPs) and identified an additional eight susceptibility loci that predispose to celiac disease [20,21]. A surprising finding from this study was that seven of the eight regions contained promising candidate genes expressed in leucocytes of the immune system. This suggested the feasibility of genetical genomics approaches using peripheral blood to assess the biological function of the celiac disease associated risk variants. One goal of genetical genomics is to uncover previously unknown biological pathways. If genetic variation affects the expression of a gene in *trans*, this suggests a biological relationship exists between the two loci. To assess this, considerable amounts of effort have been devoted to the development and application of statistical frameworks that are capable of detecting these *trans-*eQTLs. However, detection of *trans-*eQTLs in human populations has proven less successful than in mouse, rat and plant recombinant inbred lines [13,22]. It has been suggested that the extensive genetic and environmental diversity between human individuals masks many of the existing *trans-*effects.

We show that numerous *cis*-eQTLs can be identified through an expression analysis of peripheral blood RNA. We also show many of these are only detectable in peripheral blood RNA, and not in EBV-transformed B cell lines. Through a meta-analysis of these two datasets we identified numerous additional *cis*-eQTLs. We show that for some *cis-*eQTLs genetic variation does not lead to overall gene expression changes, but rather leads to shifts in the types of different splice isoforms that are produced. The complex nature of genotypic effects on gene expression limits our ability to fully elucidate the effect on RNA expression level or sequence and combined with the significant multiple testing problems prevented the accurate identification of *trans *effects.

## Methods

### Study population

115 UK celiac disease individuals were recruited from Barts and the London NHS Trust and the Oxford Radcliffe Hospitals NHS Trust after informed consent and with ethical approval. Individuals all had a small bowel endoscopic biopsy diagnosis of celiac disease, median age of 51 (23–88), a median age at diagnosis of 42 (1–75), a male to female sex ratio of 1:3, and a median length of treatment on a gluten free diet of 9.4 years (1–47). Celiac individuals responding to a gluten free diet typically show no detectable inflammation (which we confirmed at the mRNA level in peripheral blood). We also enrolled 22 healthy unrelated UK controls, with a male to female sex ratio of 1:1.2, but of unknown age.

### PAXgene RNA Extraction

2.5 ml of peripheral blood was collected into a PAXgene tube (Becton Dickinson, UK, 762165). PAXgene vials were chosen to prevent density gradient centrifugation, immortalization or *in vitro *cell culture artifacts changing mRNA profiles.

PAXgene tubes were mixed gently and incubated at room temperature for two hours. After collection, tubes were frozen at -20°C for at least 24 hours followed by storage at -80°C. RNA was isolated using the PAXgene Blood RNA isolation kit (Qiagen, UK, 762174). RNA was quantified using the Nanodrop (Nanodrop Technologies, USA). Total RNA integrity was analyzed using an Agilent Bioanalyzer (Agilent Technologies, USA).

### Anti-sense RNA synthesis, amplification, purification and hybridization to Illumina expression chips

Anti-sense RNA was synthesized, amplified and purified using the Ambion Illumina TotalPrep Amplification Kit (Ambion, USA) following the manufacturers' protocol. Complementary RNA was hybridized to Illumina HumanRef-8 v2 Whole Genome BeadChips and scanned on the Illumina BeadArray Reader. Data was handled through the Illumina BeadStudio Gene Expression module v3.2. All gene expression (MIAME compliant) and genotype data has been made freely available by submission to GEO under GSE11501.

### Quality control

Five celiac disease samples were excluded from subsequent analysis due to poor median probe intensity correlation with all other samples or incorrect sex assignment, based on an analysis of all the probes that mapped to the non-pseudoautosomal region of chromosome Y. A dataset of 110 celiac disease patients and 22 controls was then used for analysis.

### Normalization

Expression probes were mapped to the cDNA sequence from Ensembl v45_36g [23] and the NCBI build 36 genome assembly if necessary. Probes that had less than 96% sequence homology or that mapped to multiple loci were removed. Subsequent analyses were confined to autosomal probes, in order to prevent sex specific effects on gene expression. After removal of probes that map to sex chromosomes, data was quantile-quantile normalized [24].

### Celiac disease sample genotypes

All celiac disease patients were genotyped as previously described [20] using Illumina Infinium HumanHap300v1.0 BeadChips.

### Peripheral blood eQTL association analysis and false discovery rate control

257,013 autosomal SNPs were tested for association with expression levels in the 110 celiac disease samples that met analysis criteria of minor allele frequency (MAF) > 0.1, exact Hardy-Weinberg equilibrium P-Value > 0.0001 and call-rate > 0.95. Analyses were confined to those probe-SNP pairs for which the distance from probe genomic midpoint to SNP genomic location was less than 250 kb or 500 kb, depending on the analysis performed. To prevent spurious associations due to outliers, a non-parametric Spearman's rank correlation analysis was performed. In order to correct for multiple testing we controlled the false discovery rate (FDR) [25]. The distribution of all the observed p-values was used to calculate the FDR, by comparing this distribution to a null-distribution, obtained from an identical analysis where the expression phenotypes, relative to the genotypes had been permuted. Through 1,000 permutations the Spearman's rank correlation P-value threshold could be determined that corresponded to an FDR of 0.01 or 0.05.

### Validation panel: HapMap CEU samples

We compared the identified *cis-*eQTLs in the celiac peripheral blood dataset to a published human genetical genomics dataset [3]. We reanalyzed expression data from EBV-transformed B cell lines (further described as HapMap B cell line dataset) for 90 CEU HapMap samples [3]. Analyses were performed as described for the celiac disease samples. To enable a comparison between the celiac peripheral blood dataset and the HapMap B cell line dataset, only SNPs were tested that had been successfully called within HapMap and that were present on the Illumina HumanHap300 platform (257,013 SNPs). Although this is only a subset of all the SNPs that have been called for these HapMap samples, this subset of SNPs is known to capture most genetic Caucasian variation well [26].

### Analysis of over- and underrepresented biological processes and function

We investigated over- or underrepresentation of certain biological processes or functions through an analysis of all significant *cis-*eQTL genes using the Panther Classification System [27] (Binomial P-Value, Bonferroni corrected).

### Co-expression analysis

Many *cis*-eQTLs have been detected in human datasets, but only a few *trans*-eQTLs have been found [1,3,8,10]. These *trans*-eQTLs imply a biological relationship between the trans-locus and the trans-gene and as such can provide valuable biological insight. If several of these were to be identified, they would allow for the reconstruction of gene networks.

A different, regularly used approach for reconstructing gene networks is by systematically assessing co-expression between pairs of genes. We performed such a co-expression analysis for each probe that comprised a *cis*-eQTL and contrasted this to an analysis where the genetic component of the *cis*-eQTL on the probe intensity level had been removed.

We first investigated whether the identified probe that comprised a *cis*-eQTLs (probe distance of 250 kb, FDR of 0.05) showed co-expression with all other probes that mapped to different chromosomes for both the celiac peripheral blood dataset and the HapMap B cell line dataset. We correlated the measured intensity levels with all other probes that mapped on different chromosomes, through a Spearman's rank correlation analysis. By applying Bonferroni correction to account for multiple testing, sets of significantly correlated transcript-pairs could be identified.

Subsequently we used partial-correlations, enabling us to redo this co-expression analysis while effectively removing the genotypic effect on the probe intensity level for the probes that constitute the significant *cis*-eQTLs. Again, by applying Bonferroni correction, sets of significantly correlated transcript-pairs could be identified.

## Results

Celiac disease is an immune mediated disease, dominated by T_H_1 cytokine response. Given the importance of tissue specific RNA profiles, we felt that peripheral blood was an appropriate medium to study to investigate celiac disease associated genetic variants. Given the known genetic contribution to disease, we enrolled treated celiac disease individuals rather than healthy controls, leading to an enrichment for celiac disease associated genetic variants and underlying causal variants [20,28]. For 110 celiac disease samples that passed quality control, both expression at 19,867 transcripts and genotype data for 257,013 SNPs, were analyzed.

### Gene expression in celiac disease versus healthy control samples

To obtain the most accurate reflection of mRNA levels in peripheral blood leukocytes, whole blood RNA was immediately fixed during venepuncture in PAXgene vials, giving a reflection of *in vivo *RNA expression from whole blood. One hundred and fifteen treated celiac patients, all of whom were successfully treated and compliant with a gluten free diet for at least six months, were enrolled. Seventy percent of celiac disease patients were female, (66.6% of adult cases diagnosed with celiac disease in the population are female [29]). In addition, 22 random healthy control samples were enrolled to obtain a background RNA expression profile, enabling us to determine whether the patients indeed did not show ongoing inflammation. In a sex matched comparison no known inflammatory disease associated cytokines, including *IFNG *[30] and *IL2 *[31] showed significantly increased expression (sex matched Wilcoxon Signed-Ranks Test P value < 0.05) in celiac versus control samples, as was expected since these patients had been treated with a gluten free diet.

### *Cis*-associations of gene expression with SNPs

Gene expression levels for 19,867 transcripts were analyzed for significant genotypic effects at 257,013 autosomal SNPs, mapping within 500 kb of the centre of the transcript probes, resulting in 1,850,599 tests. By using a Spearman's rank correlation coefficient statistic and an FDR of 0.01 or 0.05, 1,360 and 2,178 different SNP-probe effects were detected, respectively. These reflect 394 and 658 unique probes, and 1,273 and 2,035 unique SNPs, respectively (see Table 1).

**Table 1:** Summary of *cis*-eQTL findings from celiac peripheral blood and HapMap B cell line data sets

Population	**110 celiac disease samples** **(Celiac peripheral blood dataset)**				**90 Caucasian HapMap samples** **(HapMap B cell datasets)**			
Expression data	Illumina HumanRef-8 v2 Whole Genome BeadChip (19,867 mapped transcripts)				Illumina HumanRef-6 v1 Whole Genome BeadChip (44,791 mapped transcripts)			
Genotype data	Illumina Infinium HumanHap300 BeadChip (257,013 SNPs assessed)				Subset of all HapMap Genotypes present on Illumina Infinium HumanHap300 BeadChip (257,013 SNPs assessed)			
	**FDR 0.01**		**FDR 0.05**		**FDR 0.01**		**FDR 0.05**	
SNP-Probe midpoint distance	250 kb	500 kb	250 kb	500 kb	250 kb	500 kb	250 kb	500 kb
FDR Spearman's Correlation P-Value Threshold	p < 1.67 × 10^-5^	p < 6.57 × 10^-6^	p < 1.36 × 10^-4^	p < 5.47 × 10^-5^	p < 1.08 × 10^-5^	p < 4.20 × 10^-6^	p < 8.79 × 10^-5^	p < 3.47 × 10^-5^
Number of performed tests	930,456	1,850,599	930,456	1,850,599	1,913,342	3,820,148	1,913,342	3,820,148
Number of detected different SNP-probe effects	1,529 (235)	1,360 (233)	2,487 (372)	2,178 (355)	1,981 (664)	1,799 (623)	3,226 (1,068)	2,839 (988)
Number of unique probes	470 (65)	394 (56)	765 (98)	658 (84)	613 (193)	536 (177)	994 (289)	821 (255)
Number of unique genes	460 (65)	385 (56)	753 (98)	647 (84)	563 (189)	491 (174)	903 (277)	746 (244)
Number of unique SNPs	1,432 (234)	1,273 (230)	2,315 (367)	2,035 (345)	1,743 (628)	1,601 (585)	2,826 (1009)	2,464 (920)

Numbers within brackets denote the number of *cis*-eQTLs, probes or genes that are potentially due to SNPs that map within the probe and affect the hybridization efficacies of these probes.

An identical analysis was performed on publicly available EBV-transformed B cell line expression and genotype data for 90 CEU HapMap individuals [3]. Using this dataset and a SNP-probe distance of 500 kb and FDR of 0.01 or 0.05, 1,799 and 2,839 different SNP-probe effects were detected, respectively. These reflect 536 and 821 unique probes, and 1,601 and 2,464 unique SNPs, respectively (see Table 1). Details of all detected individual *cis*-eQTLs are available in Additional file 1.

### Distance between SNPs and probes that constitute the cis-eQTLs

**Figure 1:**
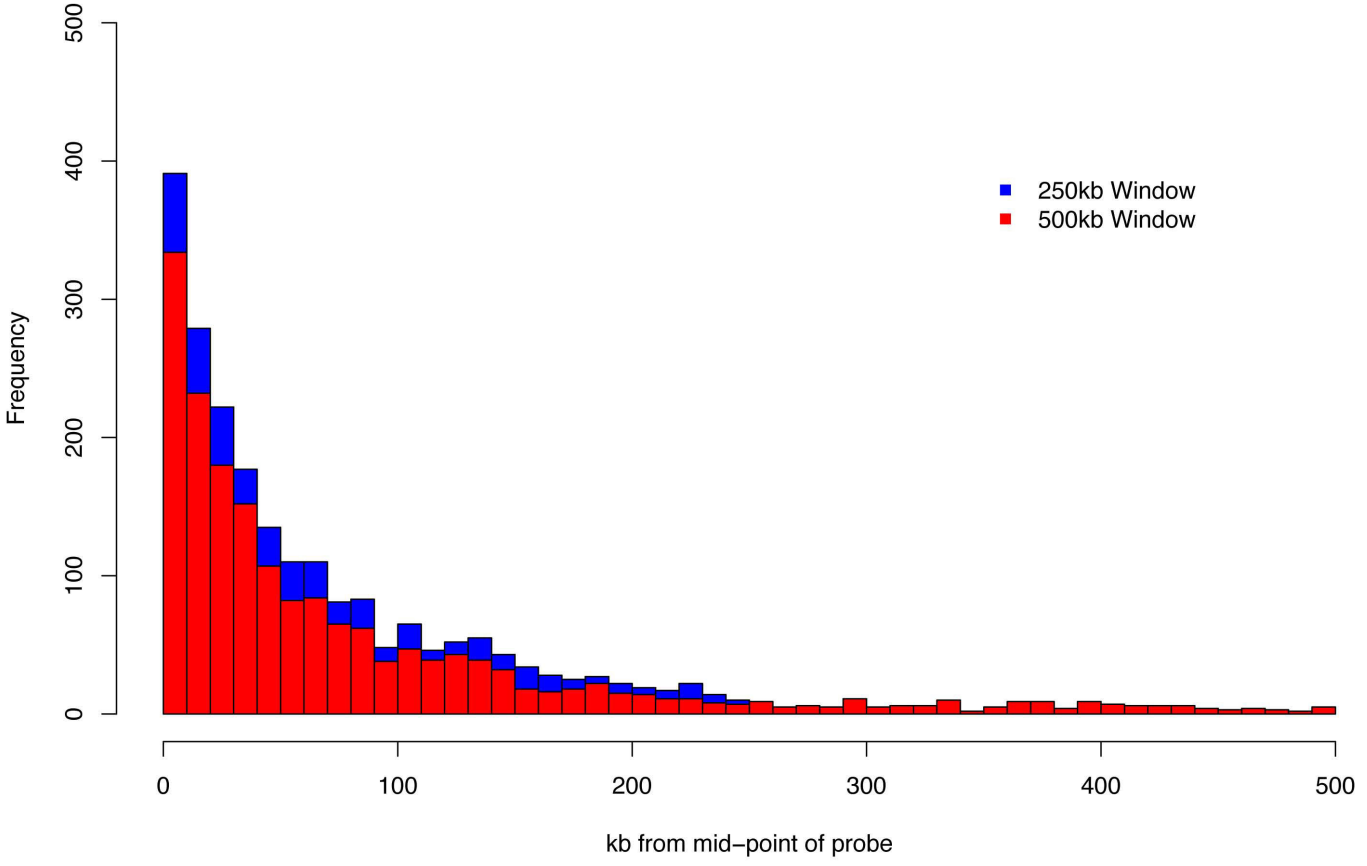
**Cumulative genomic distance distribution between SNP and probe midpoint for significant *cis*-eQTLs (FDR = 0.01, SNP-probe midpoint distance < 500 kb) in celiac peripheral blood samples**.

Our analysis was initially confined to probes that had a midpoint distance to the tested SNPs less than 500 kb. Analysis of the significant *cis-*eQTLs SNP-probe distances (Figure 1) suggests few *cis-*eQTLs have been missed by imposing this threshold, as in both datasets for 95% of the *cis-*eQTLs, the SNPs map within 250 kb of the probes. As such it is expected that an increase in statistical power can be achieved by reducing the distance to 250 kb as less tests will be performed. Indeed in the celiac peripheral blood dataset more *cis-*eQTLs were identified (in total 2,487 different SNP-probe effects were observed, compared to 2,178 in the 500 kb analysis, based on an FDR of 0.05), reflecting 765 unique probes and 2,315 unique SNPs. For the HapMap B cell line dataset 3,226 different SNP-probe effects were identified (compared to 2,839 in the 500 kb analysis, based on an FDR of 0.05), reflecting 994 unique probes and 2,826 unique SNPs (see Table 1). Subsequent analyses were confined to *cis*-eQTLs that had been detected using this 250 kb window analysis.

### Meta-analysis celiac peripheral blood and HapMap B cell lines data sets

**Figure 2:**
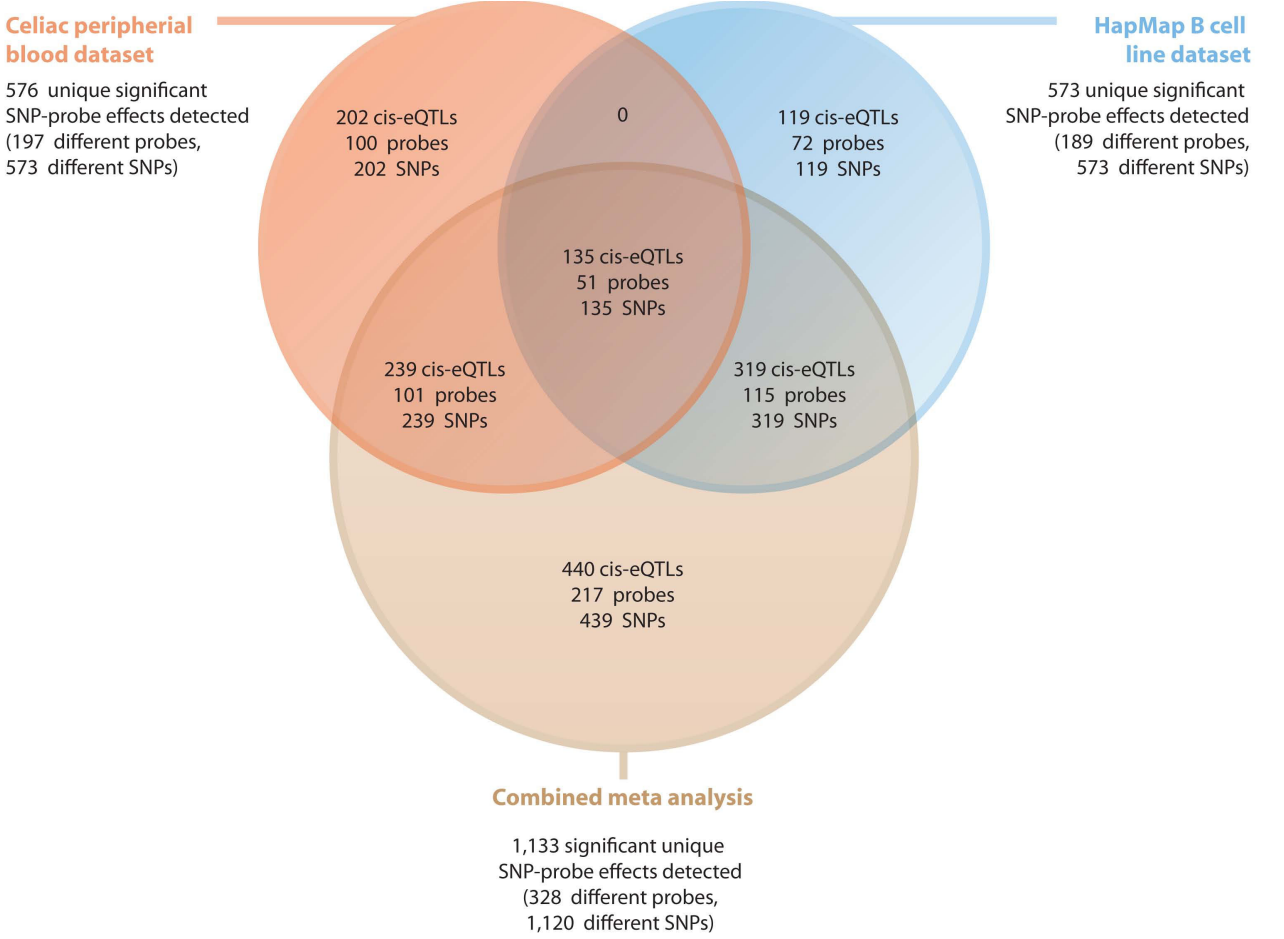
**Summary of meta-analysis of 4,681 identical probes between the celiac peripheral blood and HapMap B cell line data sets (at an FDR = 0.05, SNP-probe midpoint distance < 250 kb)**.

4,681 Illumina expression probes had oligonucleotide sequences that are shared between the two different oligonucleotide arrays used (Human Ref8 v2 & Human WG-1 v1). By limiting the analysis to a window size of 250 kb and only to SNPs that had been successfully genotyped in both studies, 576 different SNP-probe effects in the celiac peripheral blood data at a FDR = 0.05 (338 at an FDR = 0.01) were detected. These reflect 573 different SNPs and 197 different probes. In the HapMap B cell line data, 573 different SNP-probe effects were identified at an FDR = 0.05 (290 at an FDR = 0.01), reflecting 573 different SNPs and 189 different probes. A combined meta-analysis of both cohorts (weighted-Z method) identified 1,133 different SNP-probe associations at an FDR = 0.05 (428 at an FDR = 0.01) (see Figure 2). These reflect 1,120 SNPs and 328 unique probes. 440 SNP-probe pairs, reflecting 217 different probes and 439 different SNPs (FDR = 0.05) were not detected when either dataset was analyzed separately.

135 identical SNP-Probe effects (reflecting 135 different SNPs and 51 different probes) had been identified in both the 110 celiac disease samples and in the 90 HapMap B cell line samples (FDR = 0.05). In all cases, the combined meta-P-Value for each of these shared *cis-*eQTLs was more significant due to the larger sample size, indicating allelic effects in the same direction. A comparison between *cis*-eQTLs that had been detected in a large scale linkage based genetical genomics study using RNA obtained from peripheral blood mononuclear cells [2], and the *cis*-eQTLs we detected, indicates that 57.3% (distance 250 kb, 0.05FDR) of probes, displaying a genotypic effect in the celiac peripheral blood dataset, also show a linkage *cis-*eQTL signal. Of note is that *CCR3 *and *IL18RAP *are both identified as *cis*-eQTLs in both these datasets.

### Primer Polymorphisms

While it can be assumed that for most of the detected *cis*-eQTLs indeed the probe expression is affected by genetic variation, it can also be that SNPs, mapping to regions to which the probe hybridizes, may affect hybridization efficacies and result in *cis-*eQTLs [32] that are not due to expression differences. For the celiac peripheral blood dataset, 10.0% of all Illumina HumanRef-8 v2 probes map to regions that contain known dbSNP polymorphisms. For the HapMap B cell line dataset, 20.5% of all Illumina HumanRef-6 v1 probes map to known SNPs. For the probes that make up the identified *cis*-eQTLs (distance 250 kb, FDR = 0.05) this percentage is significantly higher for both the celiac peripheral blood analysis (12,5%, Fisher's Exact test P = 0.02) and even more pronounced for the HapMap B cell line dataset (29.1%, P = 7.76 × 10^-11^). Numbers within brackets in table 1 denote the number of *cis*-eQTLs, probes or genes that are potentially due to these primer polymorphisms. If these primer polymorphisms are responsible for different hybridization efficacies, these SNPs should be in LD with the SNPs that make up the *cis*-eQTLs. We could assess this for those *cis*-eQTLs where genotype data was available within HapMap Phase II for both the SNP that makes up the eQTL and for the SNPs that map within the probe that constitutes this particular eQTL. We compared the resulting distribution of r^2 ^values to a null distribution, generated by calculating the LD between the SNP that makes up an eQTL and the SNP that mapped immediately adjacent to the primer polymorphism SNP. While the mean LD (mean r^2 ^= 0.27) in the celiac peripheral blood was not significantly higher than the mean LD in the null distribution (mean r^2 ^= 0.25, Wilcoxon Mann-Whitney P-Value = 0.86), this was observed for the HapMap B cell line dataset (mean r^2 ^= 0.46, mean r^2 ^of null distribution = 0.30, Wilcoxon Mann-Whitney P-Value = 6.3 × 10^-8^).

### Differential gene expression between tissue sample types influences *cis*-eQTL detection

**Figure 3:**
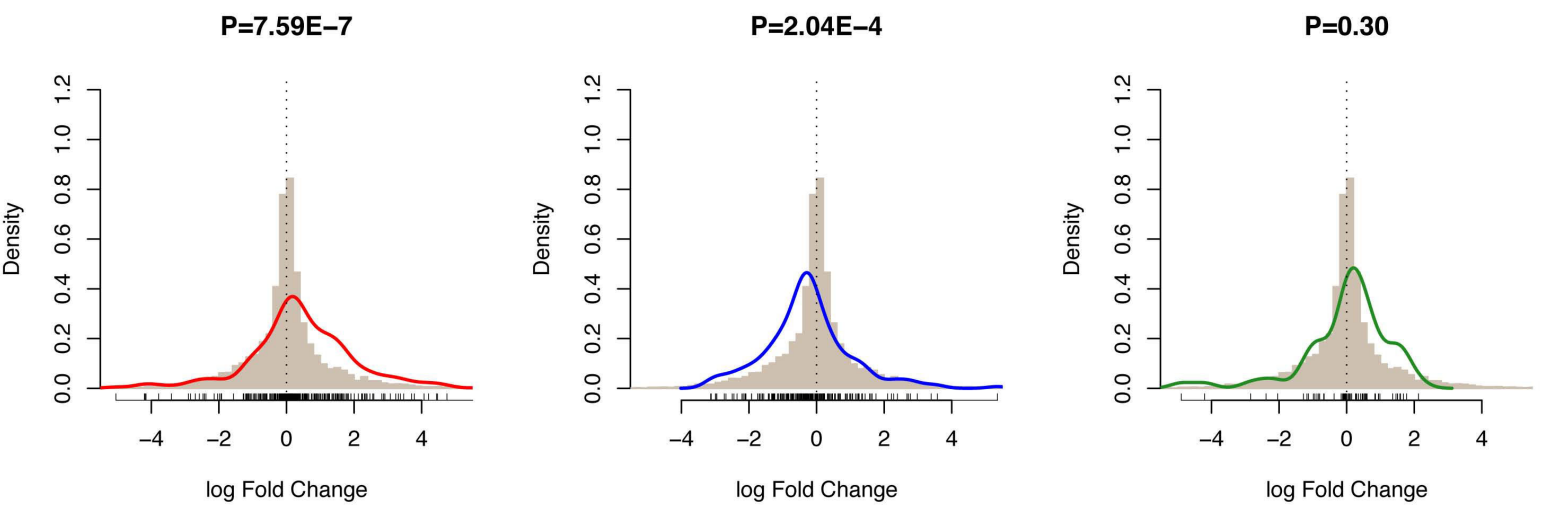
**Differential Gene Expression between tissue types results in differential *cis*-eQTL detection Differential gene expression between the celiac dataset and the HapMap dataset is represented as a histogram of log fold change**. a) Density plot of log fold change for *cis*-eQTLs detected in celiac dataset but not in HapMap samples (FDR = 0.01, SNP-probe midpoint distance < 250 kb). b) Density plot of log fold change for *cis-*eQTLs detected in HapMap dataset but not in the celiac dataset, c) Density plot of log fold change for *cis-*eQTLs detected in both data sets. P values derived from a Wilcoxon Signed-Ranks Test.

To enable a comparison of gene expression levels between the HapMap B cell line samples and the celiac peripheral blood samples, we limited our analysis to 12,401 transcripts that map to identical exons. The expression levels for these transcripts were quantile normalized. *cis-*eQTLs that have only been significantly detected in the celiac peripheral blood dataset, reflect probes that on average show higher expression in the celiac peripheral blood samples compared to HapMap B cell line samples (Wilcoxon Signed-Ranks test, P = 7.6 × 10^-7^) (see Figure 3). Conversely, probes that comprised HapMap B cell line specific *cis-*eQTLs were higher expressed than within the celiac peripheral blood dataset (P = 2.0 × 10^-4^). As expected, probes that comprised *cis-*eQTLs that were common to both data sets did not show differences in expression (P = 0.30). Noteworthy is that *IL18RAP *and *CCR3*, both recently identified as being genetically associated with celiac disease [21], exhibited significant *cis*-regulation in the celiac peripheral blood dataset but not in the HapMap B cell line dataset. *Cis*-effects for these two genes could be detected as these two genes showed markedly upregulated expression in the celiac peripheral blood dataset (expression rank order 12,072 vs. 6,431 and 11,104 vs. 3,602, respectively). These results indicate that the analysis of different cell types is valuable to gain insight in the potential functional consequences of disease associated genetic variants.

### Overrepresented biological pathways

The genes, comprising the significant *cis-*eQTLs (distance 250 kb, FDR = 0.05), showed an overrepresentation of hydrolase and transferase functions for both datasets, but 'immunity and defense' *cis*-eQTLs genes were more predominantly detected in the celiac disease peripheral blood dataset than in the HapMap B cell line dataset (see Additional file 2), as might be expected from the differential RNA profiles of the cells under investigation in each dataset.

### Significant opposite allelic directions for probes, mapping within the same genes

The observed concordance in allelic direction for the 135 significant SNP-probe effects, detected in both the 110 celiac peripheral blood and the 90 HapMap B cell line samples was perfect when probe sequences were identical. We also assessed whether the allelic directions for *cis*-eQTLs probes was the same when multiple significant *cis*-eQTL probes mapped within the same genes. We identified 14 genes (see Table 2) where this was not the case: significant *cis*-eQTL probes with different oligonucleotide sequences mapped to the same genes but showed opposite allelic directions. For three of the genes, MRPL43, OAS1 and TIPRL, we re-sequenced the probe regions in four, four and three individuals with different genotypes, respectively, and did not discover any polymorphisms, confirming that these *cis*-eQTLs do not result from artificial hybridization differences.

**Table 2:** Genes containing multiple probes that are affected by SNPs that also affect other probes in the same gene, but with opposite allelic directions

**Source**	**SNP**	**HUGO**	**Spearman**	**Probe**	Unique Illumina Identifier*	**Probe Sequence**	**Number of dbSNP polymorphisms known within probe region**
Celiac	rs1131383	POLR2J	-0.48	GI_62422568	ILMN_1657317		0
HapMap	rs1131383	POLR2J	0.57	GI_21704275	4210731		0
Celiac	rs1901198	IRF5	-0.6	GI_38683857	ILMN_1670576		0
HapMap	rs1901198	IRF5	0.5	GI_38683858	1770358		0
Celiac	rs6565724	LOC400566	0.67	GI_62177143	ILMN_1713803		0
HapMap	rs6565724	LOC400566	-0.72	GI_37544593	6040008		0
HapMap	rs6565724	LOC400566	-0.74	GI_42661283	103170403		0
Celiac	rs2863095	MRPL43	0.45	GI_28872731	ILMN_1652147		0
Celiac	rs2863095	MRPL43	-0.49	GI_28872733	ILMN_1700477		0
HapMap	rs2863095	MRPL43	0.58	GI_28872731	3940465		0
HapMap	rs2863095	MRPL43	-0.61	GI_28872733	5900487		0
HapMap	rs4768933	DIP2B	0.42	GI_17457388	101780735		0
HapMap	rs4768933	DIP2B	-0.44	GI_39930390	3940278		0
Celiac	rs10774679	OAS1	-0.54	GI_74229010	ILMN_1658247		0
Celiac	rs10774679	OAS1	0.51	GI_74229012	ILMN_1672606		0
HapMap	rs3177979	OAS1	0.63	GI_8051620	2100154		0
HapMap	rs3177979	OAS1	-0.72	GI_8051622	2100048		0
Celiac	rs1040404	TIPRL	-0.47	GI_73088904	ILMN_1779432		0
Celiac	rs1040404	TIPRL	0.5	GI_73088933	ILMN_1781457		0
HapMap	rs222851	C17orf81	-0.72	GI_44662825	6380129		0
HapMap	rs222851	C17orf81	0.64	GI_44662829	2760301		0
Celiac	rs34374	PAM	0.43	GI_21070979	ILMN_1788631	GGCTACAGTCGAAAAGGGTTTGACCGGCTTAGCACTGAGGGCAGTGACCA	0
HapMap	rs34374	PAM	-0.41	GI_21070979	60056	GCCAGTGTCTTTCTTTGGTGCCTTTCCTGTTCAGCATTCTTAGCCTGTGG	0
HapMap	rs2838859	POFUT2	-0.48	GI_34147486	5900341		1
HapMap	rs2838859	POFUT2	0.45	Hs.300736	106110059		0
Celiac	rs7084722	PTER	-0.43	GI_47933342	ILMN_1795336		0
HapMap	rs7084722	PTER	0.48	GI_20070185	5570040		1
Celiac	rs10503170	MYOM2	0.43	GI_4505314	ILMN_1716733	ATTTTCACGGGTGTGGGCACATGGGTGTGGCACCTGGACGTGTGCAGCAT	1
HapMap	rs10503170	MYOM2	-0.42	GI_4505314	6620154	TTTACACGAGGGTAGACGGCAGATGCCTGACAGAGAGTGGGTTGGCAGAC	1
Celiac	rs11680305	ADI1	-0.42	GI_8922761	ILMN_1795671	CCGGTGGTGTGATGATGCCATATACCGCAGGGCTTGCTTCTGTCAAGTGT	1
HapMap	rs11680305	ADI1	0.41	GI_8922761	4730070	GAGCTCCCACCCTAAGGGGCACACACTGAGTTGCTTATGCCACTTCCTTG	0
Celiac	rs2395185	HLA-DRB5	-0.41	GI_26665892	ILMN_1697499	GGCTCTTATTCTTCCACAAGAGAGGACTTTCTCAGGCCCTGGTTGCTACC	3
HapMap	rs2395185	HLA-DRB5	0.47	GI_26665892	450332	ACGGCCTCCCATGCATCTGTACTCCCCCTGTGTGCCACAAATGCACTACG	8

Meta-analysis of different significant but opposite allelic effects of SNPs (FDR = 0.05, SNP-probe midpoint distance < 250 kb) in the celiac peripheral blood dataset and HapMap B cell line data set. Shown are 14 genes that contain probes for either of the two platforms but that show opposite allelic directions. If probes have identical identifiers on the two platforms, but different oligonucleotide sequences, the oligonucleotide sequences are indicated.

*As provided by Illumina platform record.

### Identification of *trans *effects and conditioned co-expression analysis

We used the celiac disease dataset to assess whether significant *trans-*eQTLs could be identified (Spearman's rank correlation test P-Value < 1 × 10^-12^, FDR = 0.05). Two *trans*-eQTLs were detected: rs2318331 (mapping within *COL22A1*) with GI_48093066 (*NBPF3*) and rs12634559 (mapping downstream of *IL1RAP*) with GI_16306505 (*CASP8AP2*). However, these *trans*-eQTLs were not detected in the HapMap B cell line dataset, even after relaxing the nominal Spearman rank correlation P-value to 0.05.

**Figure 4:**
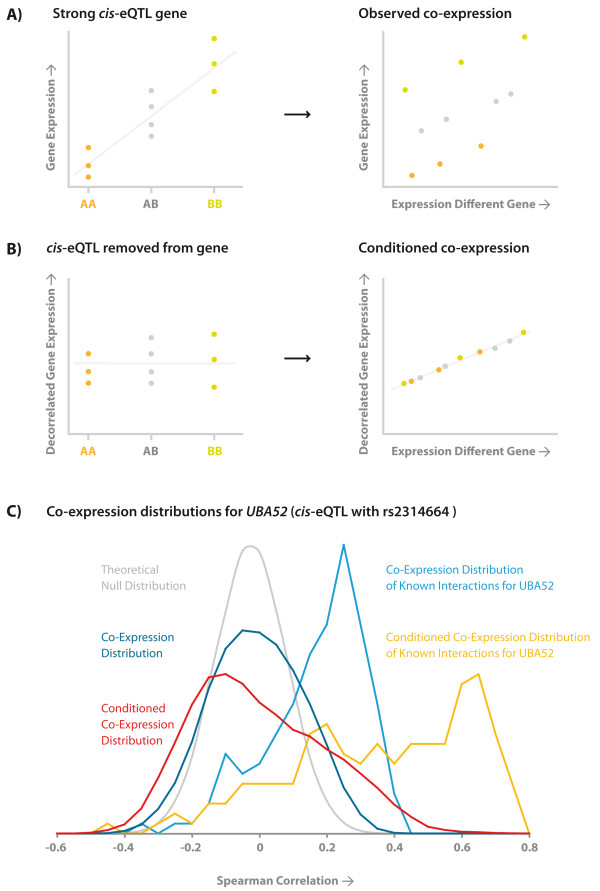
***Cis*-effects obscure detection of co-expression with other genes a) Co-expression for significant *cis*-eQTLs was determined; resulting in the identification of co-expression pairs with generally low absolute correlation coefficients b) Through removal of the genotypic effect on the *cis*-eQTL probe, for some *cis*-eQTLs strong co-expression can be more easily detected**. c) An example for *UBA52 *indicates that a conditioned co-expression analysis can help to identify meaningful biological relationships: Within the conditioned co-expression distribution (indicated in dark blue) there are more strongly co-expressed genes, opposed to an unconditioned analysis (indicated in red). This is supported by an analysis of 156 known interacting genes for *UBA52*: In the conditioned co-expression analysis (indicated in light blue) co-expression is generally much stronger than in the unconditioned analysis (indicated in yellow).

As *trans*-eQTLs implicate (direct or indirect) biological relationships, it is somewhat disappointing that none were clearly identified. An alternative way to identify biological relationships is by using co-expression analysis (although such an analysis does not have the aim to identify or explain *trans*-eQTLs). We conducted such a co-expression analysis and compared it to a co-expression analysis that also takes the genetic variation into account. First, co-expression was assessed between each of the probes that constitute *cis*-eQTLs (SNP-probe midpoint distance < 250 kb, FDR = 0.05) and all other probes (Figure 4a) that do not map to the same chromosome, by calculating all pair-wise Spearman correlation coefficients. This resulted in 13,003,505 and 42,270,807 pair-wise tests for the celiac peripheral blood dataset (using 765 different *cis-*probes) and the HapMap B cell line dataset (using 994 different *cis-*probes), respectively. Through Bonferroni correction we accounted for multiple testing, resulting in the identification of 50,821 and 168,292 significantly correlated transcript-pairs respectively (Spearman's correlation test P-Value < 0.05 after multiple testing correction).

Subsequently, we redid this co-expression analysis after having removed the genotypic effect on the probe intensity level for the probes that constitute the significant *cis*-eQTLs (Figure 4b). We observed a considerably increased number of significantly correlated transcript-pairs: 54,773 (an increase of 7.8%) and 258,874 (an increase of 53.8%), respectively.

To validate whether this increase in co-expressed transcripts reflects known biology, we assessed a collection of 80,350 known biological interactions (derived on 17 April 2007 from KEGG [33], BioGrid [34], Reactome [35], BIND [36], HPRD [37] and IntAct [38]). For the celiac peripheral blood dataset 91 of the 50,821 (0.179%) significantly correlated transcript-pairs that had been identified in the unconditioned co-expression analysis are known to interact. In the conditioned co-expression analysis 106 of the 54,773 (0.194%) transcript pairs were known to interact, indicating an 8.0% increase in the proportion of known biological interactions that was identified. Comparable results were obtained for the HapMap B cell line dataset: For the unconditioned co-expression analysis 334 of the 168,292 (0.195%) significant transcript-pairs are known to interact. In the conditioned co-expression analysis 634 of the 258.874 (0.245%) significant transcript-pairs are known to biologically interact, representing an increase of 23.4% in the proportion of known interactions among the most significantly co-expressed transcript-pairs.

Figure 4c provides an example for Ubiquitin (*UBA52*), for which a strong *cis*-eQTL was observed for probe GI_15451941 with rs2314664 (Spearman's correlation coefficient P-Value = 1.11 × 10^-16^) in the HapMap B cell line dataset. Within this probe three other polymorphism map (rs6554, rs34040670 and rs3209501), of which rs6554 is in near perfect LD with rs2314664 (r^2 ^= 0.98, HapMap CEU population), suggesting this *cis*-eQTL does not reflect a real expression difference, but rather a difference in hybridization efficacy. Co-expression with other genes is already present in an unconditioned co-expression analysis, because the distribution of co-expression for *UBA52 *with all other genes differs significantly from a theoretical null-distribution (Wilcoxon-Mann Whitney P-Value < 1 × 10^-12^). However, in the conditioned co-expression analysis, this difference is more pronounced (see figure 4c). When confining this analysis to a set of 156 known interactions for *UBA52*, overall co-expression was significantly stronger for these pairs of genes in the conditioned (mean absolute Spearman's correlation coefficient = 0.39) than in the unconditioned co-expression analysis (mean absolute Spearman's correlation coefficient = 0.20, Wilcoxon-Mann Whitney P-Value < 10^-50^).

## Discussion

We have demonstrated the use of peripheral blood RNA samples for the detection of *cis-*eQTLs and have shown that there is strong allelic concordance with *cis-*eQTLs that also had been detected in a HapMap B cell line dataset. These results indicate that a meta-analysis with larger sample size and hence statistical power results in a considerable increase in the detected *cis-*eQTL even though the arrays that had been used were different. Some *cis-*eQTLs can be observed across multiple tissue types, in all cases in the same allelic direction, suggesting a major conserved function. However most of the detected *cis-*eQTLs in these datasets were only detected in one of the two tissues, suggesting that more insight can be gained in the functional consequences of genetic variation by performing genetical genomics studies using different types of cells and tissues. This point is particularly relevant for identifying the function of risk variants for common diseases: for example study immune tissues for immune-mediated diseases, adipose tissue for obesity traits [8], brain tissue for neurological traits [10]. Genetical genomics experiments performed in outbred human populations are additionally complex and in addition to tissue specific RNA profiles, care should be taken to investigate alternative RNA sequence events, primer polymorphisms that generate false eQTLs and strong *cis*-eQTLs that obscure weaker *trans *effects.

It is attractive to assume most of the observed *cis-*eQTLs reflect overall gene expression level alterations. However, we did observe 14 genes (Table 2) where different probes showed significant opposite allelic effects. For at least five out of the 14 genes (*POFUT2, PTER, MYOM2, ADI1 *and *HLA-DRB5*) polymorphisms within the probe are known to exist in dbSNP. For each of these genes, it may be that one *cis*-eQTL for instance reflects a real expression difference, whereas the other reflects a hybridization effect due to the presence of these SNPs within the probe. However, recently Kwan *et al *[39] provided another potential explanation, as they showed for three of the 14 genes (*IRF5, MRPL43 *and *PTER*), using Affymetrix GeneChip Human Exon 1.0 ST Arrays, that different genetic variants can result in premature 3' termination events. The SNPs that make up these *cis*-eQTLs are all in strong linkage disequilibrium with the SNPs Kwan *et al *described (IRF5: rs7808907 and rs6969930: D' = 1, R^2 ^= 0.74 rs2863095, MRPL43: rs2863095 and rs12241232: D' = 0.89, R^2 ^= 0.75, PTER: rs7909832 and rs1055340: D' = 1, R^2 ^= 1), supporting our observations. Kwan *et al *examined different exonic effects through an independent validation using quantitative RT-PCR (out of a total of 25 validated genes) and estimated that only 39% of the detected *cis-*eQTLs influence overall gene expression levels. For the remaining *cis-*eQTLs genetic variation results in preliminary terminated transcripts (18%), not initiated transcripts (11%), transcripts that are spliced differentially (26%) or a combination of these (6%). We acknowledge that our use of oligonucleotide arrays, predominantly targeting the 3' end of genes, gives a more limited picture of splicing since only 3' termination events can be seen. However, the data presented above and alluded to by Kwan *et al *[39] suggest that SNP genotypes can have a significant effect on alternatively spliced transcript isoforms. This additional layer of complexity should be examined in future genetical genomics experiments to fully elucidate the genotypic effects on RNA.

Although the two *trans*-effects identified (*IL1RAP *and *CASP8AP2*) are interestingly both involved in an apoptosis pathway, these *trans*-eQTLs were not detected in the HapMap B cell line dataset. These *trans*-eQTL results are in strong contrast to the number of *cis*-eQTLs we detected within the two data sets. This results primarily from the limited statistical power to detect *trans*-eQTLs, due to the number of statistical tests that need to be performed. Additionally, in order to cause a *trans*-effect, more intermediate biological steps are required that introduces additional biological noise, lessening the correlation between genotype and *trans*-gene expression. *Cis*-eQTLs can have a significant impact on the co-expression of genes; this genotypic variation may hinder the identification of significant *trans *effects. This adds an additional layer of complexity to the analysis of genotypic effects on gene expression in outbred populations.

As it has been one of the goals of genetical genomics to identify biological relationships we suggest that the conditioned co-expression analysis we carried out here, might help to uncover these: We have shown that more known biological relationships can be identified when using genetical genomics to perform co-expression analyses that have been conditioned on genotype. An explanation for this observation is that cis-eQTLs sometimes convolute co-expression, as is exemplified for *UBA52*, where primer polymorphisms probably affect hybridization characteristics.

## Conclusion

This study shows that PAXgene isolated peripheral blood RNA is a powerful resource for investigating functional consequences of genetic variation. We have shown that for some of the *cis-*eQTLs the functional consequences are more complex than previously assumed. Additionally, these findings imply that biological relationships can be extracted in outbred populations, although in a somewhat different manner than what is commonly used to detect biological relationships through *trans*-eQTLs in inbred model organisms.

As this study has only combined genetics with genomics, we envision more extensive integrative approaches, incorporating *e.g. *epigenetics and proteomics, will help to improve the detection of previously unknown biological pathways.

## Abbreviations

SNP: Single Nucleotide Polymorphisms; eQTL: Expression Quantitative Trait Locus

## Competing interests

The authors declare that they have no competing interests.

## Authors' contributions

LCD and DVH collected patient samples. GH extracted RNA and performed microarray experiments assisted by MB. LF, RCJ, GH, CW and DVH designed the experiment. KH undertook the genotyping. LF and GH performed the data analysis assisted by RCJ and MAS. Sequencing was carried out by GT. GH and LF drafted the manuscript. All authors read and approved the final manuscript.

## Pre-publication history

The pre-publication history for this paper can be accessed here:



## Supplementary Material

Additional file 1 **All detected *cis*-eQTLs. All detected *cis*-eQTLs in celiac peripheral blood and HapMap datasets 250 kb and 500 kb from the hybridization probe midpoint** Click here for file

Additional File 2 **Biological processes and functions for the detected *cis*-eQTLs**. Listed are significantly over- and underrepresented biological processes and functions for the detected *cis*-eQTLs (FDR = 0.05, SNP-probe midpoint distance < 250 kb), derived through the Panther Classification System (Binomial test, Bonferroni corrected) Click here for file
